# Intracranial aneurysms formation after radiotherapy for head and neck cancer: a 10-year nationwide follow-up study

**DOI:** 10.1186/s12885-019-5766-2

**Published:** 2019-06-04

**Authors:** Wei-Hsun Yang, Yao-Hsu Yang, Pau-Chung Chen, Ting-Chung Wang, Ko-Jung Chen, Chun-Yu Cheng, Chia-Hsuan Lai

**Affiliations:** 1 Division of Neurosurgery, Department of Surgery, Chang Gung Memorial Hospital, Chia-Yi, Taiwan; 2 Department of Traditional Chinese Medicine, Chang Gung Memorial Hospital, Chia-Yi, Taiwan; 3 Health Information and Epidemiology Laboratory of Chang Gung Memorial Hospital, Chiayi, Taiwan; 4 School of Traditional Chinese Medicine, College of Medicine, Chang Gung University, Taoyuan, Taiwan; 5 Institute of Occupational Medicine and Industrial Hygiene, National Taiwan University College of Public Health, Taipei, Taiwan; 6 Department of Environmental and Occupational Medicine, National Taiwan University Hospital and National Taiwan University College of Medicine, Taipei, Taiwan; 7 Department of Radiation Oncology, Chang Gung Memorial Hospital, No 6, West Section, Chia-Pu Road, Putz City, Chia-Yi, Taiwan 613

**Keywords:** Vasculopathy, Radiotherapy, Aneurysm, Head and neck cancer

## Abstract

**Background:**

Intracranial aneurysms after radiotherapy (RT) have previously been reported. However, the majority of studies were case reports. Therefore, we performed a nationwide study to explore the risk of radiation-induced intracranial aneurysms.

**Methods:**

This study included patients diagnosed with head and neck cancer (ICD9: 140–149, 161). Intracranial aneurysms formation was identified using the following ICD9 codes: nonruptured cerebral aneurysm (ICD9:4373), aneurysm clipping (ICD9:3951). Patients who did not receive curative treatment and those with intracranial aneurysms before the diagnosis of head and neck cancer were excluded.

**Results:**

In total, 70,691 patients were included in the final analysis; they were categorized into the following three groups: nasopharyngeal carcinoma (NPC) with RT, non-NPC with RT, and non-NPC without RT. Patients in the NPC with RT group had the highest risk of developing intracranial aneurysms (hazard ratio (HR) 2.57; *P* <  0.001). In addition, hypertension was also a risk factor of developing intracranial aneurysms (HR 2.14; *P* <  0.01). The mean time interval from cancer diagnosis to intracranial aneurysm formation in the NPC with RT group was 4.3 ± 3.1 years.

**Conclusions:**

Compared with the non-NPC with RT and the non-NPC without RT groups, patients with NPC who received RT had a higher risk of developing intracranial aneurysms.

## Background

Over the last century, the average life expectancy of cancer patients has increased, owing in large part to the increase in the application of radiotherapy (RT) and improvements in healthcare. With this increase in lifespan, the long-term effects of RT have become more important. Among the variety of delayed complications that occur after RT, radiation-induced vascular diseases have previously been studied [1–6]. However, the majority of studies have focused on the risk of intracranial occlusive stroke [7, 8] and of intracranial atherosclerosis [9].

Intracranial aneurysms formation after RT have been reported since 1967 [10, 11], usually in case reports [12–22]. In 2000, Maruyama et al. performed a literature review that included both radiation-induced aneurysms and moyamoya vessels [23]. They concluded that radiation-induced vasculopathy was rare, but that it may be fatal. In 2014, Nanney et al. performed a literature review that focused on radiation-induced intracranial aneurysms [24]. A total of 46 patients with 69 intracranial aneurysms between 1978 and 2013 were included, and details concerning the pathologic features of the aneurysms, including fibrosis, necrosis, atherosclerotic changes, and inflammation of the aneurysmal wall, were described. However, their study had several limitations, including a limited number of cases, inconsistent diagnostic criteria, and a lack of reliable histologic and radiographic characteristics to establish a statistical model.

The association between RT and intracranial aneurysms formation continues to be debated due to varying opinions concerning the effects of radiation on vessel walls. Indeed, while previous studies demonstrated weakened vessels after radiation, Mecermott et al. [25] showed vessel thickening, which was also noted by another study [6, 26]. Similar results were also demonstrated in other studies, which showed intimal hyperplasia, adventitia thickening, and increased connective tissue production after radiation [10, 27–32]. However, in an article investigating the outcome of 1400 patients with arteriovenous malformation treated with radiosurgery [33, 34], no intracranial aneurysms developed during an 8-year follow-up period. Therefore, the relationship between RT and intracranial aneurysms formation should be clarified.

The National Health Insurance Research Database (NHIRD) was provided by the National Health Research Institute in Taiwan. This database contains the medical claim records of 26 million individuals from 1996 to 2009, which includes almost 97% of the population of Taiwan. This database not only documents the type of cancer a patient presents, but also describes the treatment modalities used and provides long-term follow-up records. Patients with head and neck cancer have been reported to have thickened intimas and an increased rate of atherosclerotic plaque formation after RT [35]. In a study by Huang et al. that used the NHIRD, a higher risk of stroke after RT and chemotherapy was found in young patients with head and neck cancer [1]. In this study, we aimed to evaluate the risk of intracranial aneurysmal development in patients with head and neck cancer.

## Methods

### Data source

Data were sourced from the NHIRD. The National Health Insurance (NHI) is a compulsory universal program for all residents of Taiwan, and the NHIRD is a comprehensive healthcare database that covers nearly the entire population of Taiwan. Information concerning admissions and outpatient visits including information on patient characteristics such as sex, date of birth, date of admission, date of discharge, dates of visits, and up to five discharge diagnoses or three outpatient visit diagnoses were obtained from the database. Diagnoses were made using the International Classification of Diseases, Ninth Revision, Clinical Modification (ICD-9-CM) codes.

In this study, the Registry of Catastrophic Illness Patient Database (RCIPD) from the NHIRD was used. The RCIPD contains the medical records of all confirmed cases of catastrophic illness, including cancers, end-stage renal disease, congenital anomalies, autoimmune diseases, etc. Patients diagnosed with cancer can apply for catastrophic illness certification, and the Bureau of the NHI performs rigorous validation of all cancer diagnoses, during which at least two independent specialists review the medical, laboratory, histological, and imaging records of each patient.

### Ethics statement

This study adhered to strict confidentiality guidelines in accordance with regulations regarding personal electronic data protection. It was approved by the ethics review board in our institution (No. 201700121B1). All study participants were above the legal age of consent for research of eighteen years old. All data were analyzed anonymously; the requirement for informed consent was waived by the institutional review board.

### Study subjects

This study included both inpatients and outpatients diagnosed with head and neck cancer (ICD9: 140–149, 161) during 2000–2012. Patients with intracranial aneurysms prior to the diagnosis of head and neck cancer or patients with missing data were excluded. Nasopharyngeal carcinoma (NPC) patients without RT, other head and neck cancer patients who did not received surgery nor RT, patients had delayed treatment longer than three months and the patients had the prior cancer history were also excluded. All study subjects were categorized into three groups as follows: 1) NPC patients with RT (NPC-RT); 2) other head and neck cancer patients with RT (non-NPC-RT); and 3) other head and neck cancer patients without RT (non-NPC-NRT). The group of non-NPC-NRT group were used as control groups. All medical records of the study cohort during 1997–2013 were extracted and analyzed, and all enrolled study subjects were followed until the diagnosis of intracranial aneurysms, death, or the end of 2013.

Intracranial aneurysms were identified using the following ICD9 codes: cerebral aneurysm, nonruptured (ICD9: 4373). Patients who underwent aneurysm clipping (ICD9:3951) were also defined as having intracranial aneurysms. However, patients with intracranial spontaneous subarachnoid hemorrhage (SAH) and were in a deep coma (Glasgow Coma Scale less than six) may not receive imaging studies such as angiography or computed tomography (CT) angiography due to unstable vital signs. Therefore, these patients were not diagnosed with intracranial aneurysms. In addition, a patient diagnosed with an aneurysm may be treated with endovascular coiling instead of surgical clipping in recent years. Unlike surgical clipping, no appropriate ICD-9 codes exist for coiling. Therefore, we were unable to include patients treated with coiling or other endovascular techniques in this study.

### Treatments in NPC and non-NPC head and neck cancer patients

The treatment of NPC patients was mainly radiotherapy with or without chemotherapy based on different clinical stages [36]. Most hospitals in Taiwan treated NPC patients with a uniform RT dose that ranged from 66 to 72 Gy [37–39].

For oral cavity cancer patients, surgery played the most important role in multidisciplinary treatments in Taiwan [36]. Most patients with resectable disease received radical surgery followed by adjuvant RT or CCRT if there were high risk factors such as positive surgical margins, extranodal extension, pathological T3–4 primary, N2–3 nodal disease, perineural invasion or lymphovascular invasion. The adjuvant RT dose ranged from 60 to 66 Gy. For those patients with unresectable disease, definitive concurrent chemoradiotherapy (CCRT) with RT dose 66–72 Gy was usually used.

For oropharyngeal or hypopharyngeal cancer patients in Taiwan, most common treatments were radiotherapy with or without chemotherapy because patients usually favor organ preservation strategies [36]. The RT dose was 66–72 Gy for definitive CCRT. About 30 % of these patients received surgical intervention [36]. Adjuvant RT or CCRT with RT dose 60–66 Gy was usually given if there were high risk factors such as positive surgical margins, extranodal extension, pathological T3–4 primary, N2–3 nodal disease, perineural invasion or lymphovascular invasion.

### Statistical analysis

The Kaplan–Meier method was used for univariate analysis and the log-rank test was used to detect differences. Cox proportional hazards model and competing risk analysis were used for multivariate analysis to evaluate the association between RT and intracranial aneurysms formation. Hazard ratios (HRs) and 95% confidence intervals (CIs) were computed after adjusting for comorbidities and sociodemographic characteristics (age, sex, income, and level of urbanization). All analyses were performed using the SAS ver. 9.4 software (SAS Institute, Cary, NC, USA).

## Results

### General characteristics

**Fig. 1:**
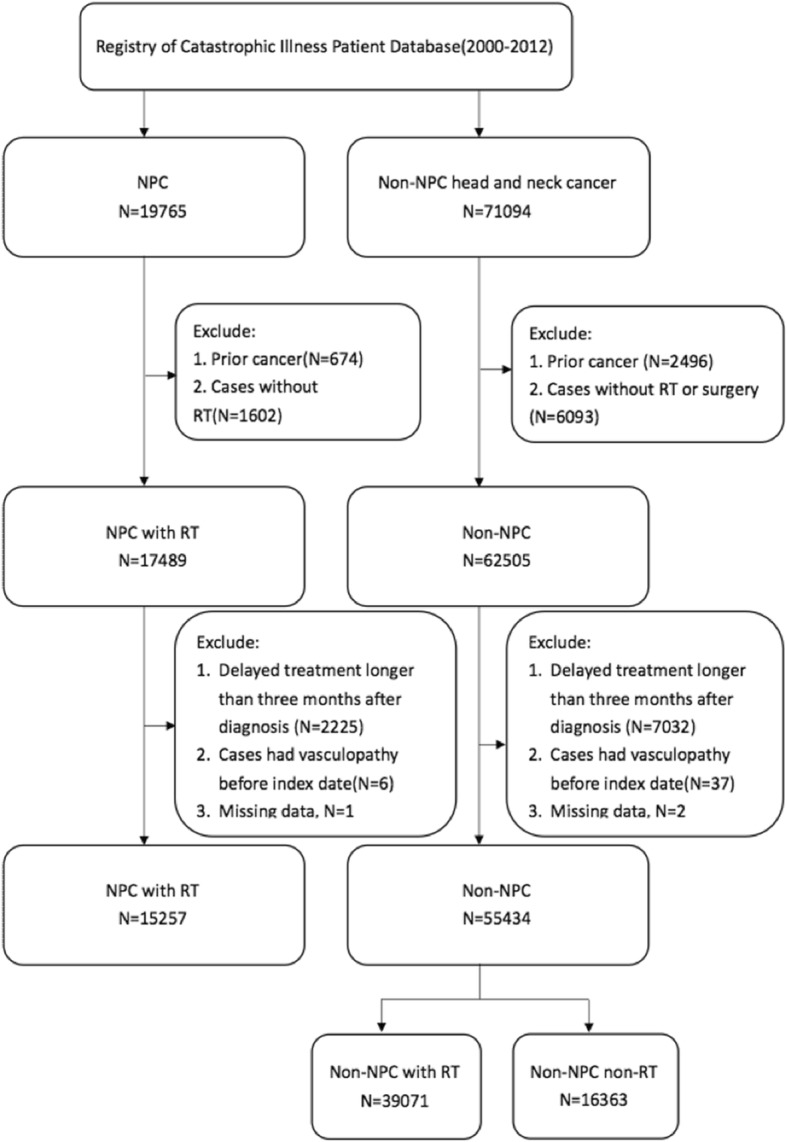
Flow diagram of this nationwide-based study. Abbreviations: NPC-RT, nasopharyngeal cancer patients with radiotherapy; Non-NPC–RT, other head and neck cancer patients with radiotherapy; Non-NPC-NRT, other head and neck cancer patients without radiotherapy

During 2000–2012, 70,691 head and neck patients met the inclusion criteria were included in the final analysis (Fig. 1). There were 15,257, 39,071, and 16,363 subjects in the NPC-RT, non-NPC-RT, and non-NPC-NRT groups, respectively (Table 1).

**Table 1:** Baseline characteristics of the NPC-RT, Non-NPC-RT, and Non-NPC-NRT groups

Variables	NPC-RT (*n* = 15,257)		Non-NPC–RT (*n* = 39,071)		Non-NPC-NRT (*n* = 16,363)		*P* value
	n	%	n	%	n	%	
Gender							< 0.0001
Male	11,443	75.00	35,958	92.03	14,500	88.61	
Female	3814	25.00	3113	7.97	1863	11.39	
Age, years							< 0.0001
< 45	5302	34.75	8250	21.12	3530	21.57	
45–65	7949	52.10	23,536	60.24	9265	56.62	
> 65	2006	13.15	7285	18.65	3568	21.81	
Comorbidities							
Arrhythmia							< 0.0001
Yes	1617	10.6	4361	11.16	2144	13.10	
No	13,640	89.40	34,710	88.84	14,219	86.90	
HTN							< 0.0001
Yes	5845	38.31	16,169	41.38	8166	49.91	
No	9412	61.69	22,902	58.62	8197	50.09	
DM							< 0.0001
Yes	2694	17.66	9093	23.27	4773	29.17	
No	12,563	82.34	29,978	76.73	11,590	70.83	
COPD							< 0.0001
Yes	2489	16.31	7198	18.42	3134	19.15	
No	12,768	83.69	31,873	81.58	13,229	80.85	
Dyslipidemia							< 0.0001
Yes	3837	25.15	9137	23.39	5398	32.99	
No	11,420	74.85	29,934	76.61	10,965	67.01	
Chronic Kidney disease							< 0.0001
Yes	647	4.24	1728	4.42	879	5.37	
No	14,610	95.76	37,343	95.58	15,484	94.63	
Coronary artery disease							< 0.0001
Yes	2435	15.96	6599	16.89	3742	22.87	
No	12,822	84.04	32,472	83.11	12,621	77.13	
Heart failure							< 0.0001
Yes	695	4.56	2219	5.68	1150	7.03	
No	14,562	95.44	36,852	94.32	15,213	92.97	
Liver cirrhosis							< 0.0001
Yes	443	2.90	3588	9.18	1053	6.44	
No	14,814	97.10	35,483	90.82	15,310	93.56	
Chemotherapy							< 0.0001
Yes	12,596	82.56	27,637	70.74	1469	8.98	
No	2661	17.44	11,434	29.26	14,894	91.02	
Intracranial aneurysms							
Yes	40	0.26	36	0.09	9	0.06	< 0.0001
No	15,217	99.74	39,035	99.91	16,354	99.94	
Death	5014	32.86	21,796	55.79	3548	21.68	< 0.0001

Abbreviations: *NPC-RT* nasopharyngeal cancer patients with radiotherapy, *Non-NPC–RT* other head and neck cancer patients with radiotherapy, *Non-NPC-NRT* other head and neck cancer patients without radiotherapy, *HTN* hypertension, *DM* diabetes mellitus, *COPD* chronic obstructive pulmonary disease

Among these enrolled subjects, 40 NPC-RT cases were diagnosed with intracranial aneurysms after RT. In Non-NPC-RT and Non-NPC-NRT groups, there were 36 cases and 9 cases diagnosed with intracranial aneurysms during follow-up period. Compared to Non-NPC-RT and Non-NPC-NRT groups, the NPC-RT group had higher risk of developing intracranial aneurysms (*P* <  0.0001).

### General characteristics of patients with newly diagnosed intracranial aneurysms

**Fig. 2:**
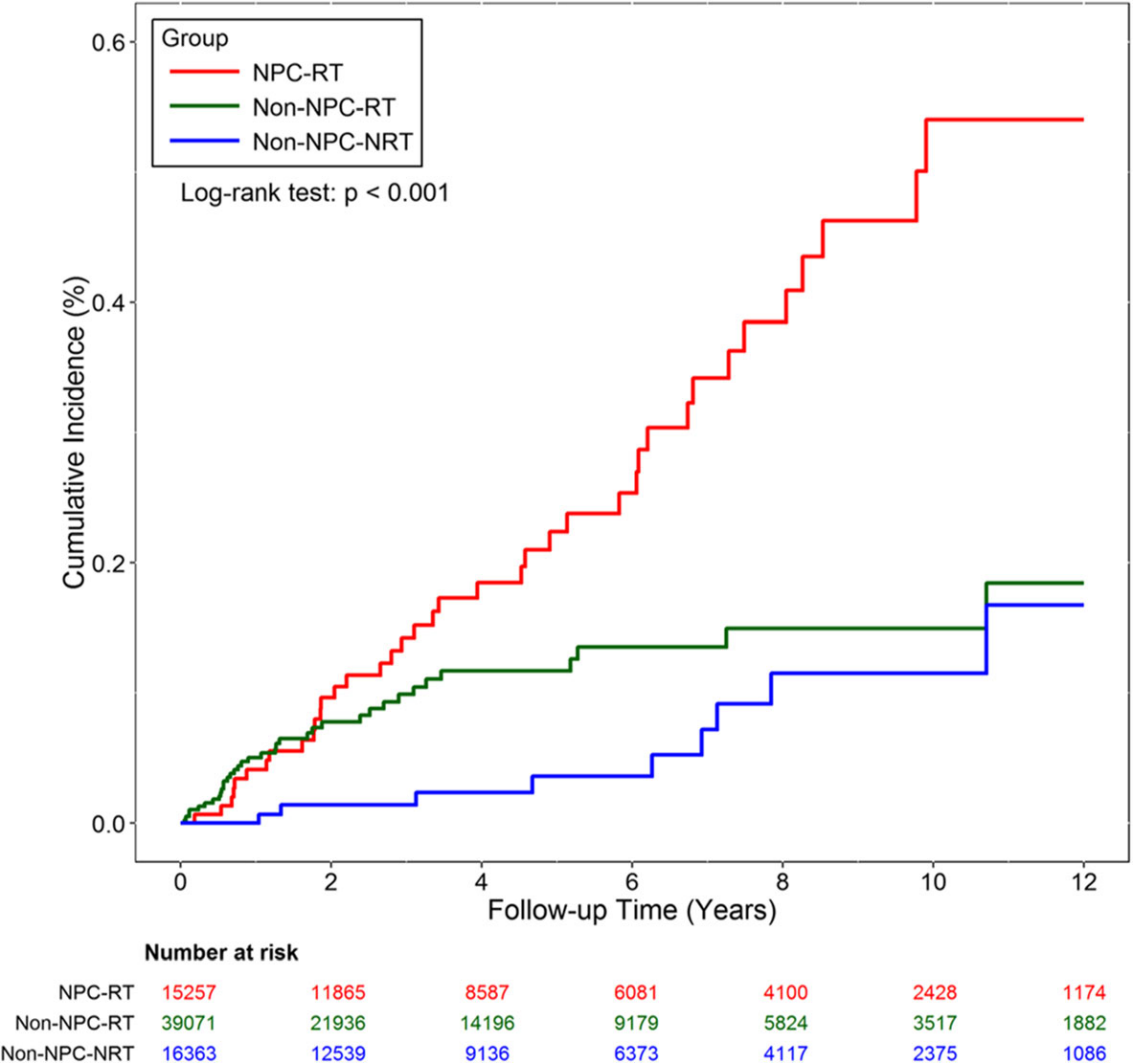
Cumulative incidence of aneurysm formation among the three groups

The incidence of intracranial aneurysms formation was 47.8, 23.9, and 10.4 per 100,000 person-years in the NPC-RT, non-NPC-RT, and non-NPC-NRT groups, respectively (Table 2). The NPC-RT group had a significantly higher incidence of newly formed intracranial aneurysms compared with the other two groups (*P* <  0.0001; Fig. 2). Although the incidence of new intracranial aneurysms was higher in the non-NPC-RT group compared with the non-NPC-NRT group, the difference was not statistically significant (*P* = 0.57).

**Table 2:** Characteristics of patients with radiation-induced intracranial aneurysms

Variables	NPC-RT	Non-NPC-RT	Non-NPC-NRT
Number	40	36	9
Incidence, 100,000 persons-year (95% CI)	47.8 (35.1–65.2)	23.9 (17.2–33.1)	10.4 (5.4–19.9)
Interval time, years (± SD)	4.3 (±3.1)	1.9 (±2.2)	5.5 (±3.2)
Mean age, years (± SD)	50.7(±11.3)	56.7 (±9.4)	53.8 (±13.7)
Gender			
Male (%)	31 (77.5)	28 (77.8)	9 (100.0)
Female (%)	9 (22.5)	8 (22.2)	0 (0)

Abbreviations: *NPC-RT* nasopharyngeal cancer patients with radiotherapy, *Non-NPC–RT* other head and neck cancer patients with radiotherapy, *Non-NPC-NRT* other head and neck cancer patients without radiotherapy, *CI* confidence interval, *SD* standard deviation

Among the patients with newly formed intracranial aneurysms, 77.5, 77.8, and 100.0% were male in the NPC-RT, non-NPC-RT, and non-NPC-NRT groups, respectively. The mean ages were 50.7 ± 11.3, 56.7 ± 9.4, and 53.8 ± 13.7 years, respectively, and the time intervals from aneurysm formation to treatment were 4.3 ± 3.1, 1.9 ± 2.2, and 5.5 ± 3.2 years, respectively.

### Factors associated with newly developed intracranial aneurysms

Multivariate regression analysis revealed that RT was a significant risk factor for intracranial aneurysms development (Table 3). The risk of intracranial aneurysms formation was highest in the NPC-RT group (HR = 1.99, 95% CI: 1.23–3.21, *P* <  0.01) and lowest in the non-NPC-NRT group (HR = 0.60, 95% CI:0.26–1.35, *P* = 0.21). Moreover, hypertension was also associated with an increasing risk of aneurysm development (HR = 2.01, 95% CI: 1.22–3.31, *P* <  0.01).

**Table 3:** Cox proportional hazards model and competing risk analyses

Variables	Aneurysms formation					
	Adjusted			Competing risk analysis		
	HR	95%CI	*P* value	HR	95%CI	*P* value
Group						
NPC-RT	1.99	1.23–3.21	< 0.01	2.57	1.55–4.24	< 0.001
Non-NPC-NRT	0.60	0.26–1.35	0.21	0.70	0.32–1.56	0.38
Non-NPC-RT	1.00	reference		1.00	reference	
Gender						
Male	0.77	0.44–1.34	0.35	0.71	0.39–1.28	0.26
Female	1.00	reference		1.00	reference	
Age						
< 45	1.00	reference		1.00	reference	
45–65	1.20	0.69–2.06	0.52	1.07	0.64–1.80	0.79
> 65	0.97	0.43–2.19	0.94	0.68	0.32–1.46	0.32
Comorbidity						
Arrhythmia	1.08	0.56–2.07	0.82	1.07	0.57–2.02	0.83
HTN	2.01	1.22–3.31	< 0.01	2.14	1.34–3.43	< 0.01
DM	1.04	0.61–1.78	0.87	0.99	0.59–1.66	0.97
COPD	1.07	0.62–1.85	0.80	1.12	0.63–2.00	0.69
Dyslipidemia	0.88	0.53–1.46	0.61	0.98	0.60–1.58	0.92
CKD	0.93	0.33–2.58	0.88	0.92	0.32–2.64	0.87
CAD	1.51	0.88–2.61	0.14	1.59	0.92–2.74	0.10
Heart failure	0.32	0.08–1.34	0.12	0.29	0.07–1.20	0.09
Liver cirrhosis	1.41	0.61–3.28	0.43	1.15	0.48–2.73	0.76
Chemotherapy						
Yes	1.71	0.97–3.00	0.06	1.37	0.83–2.26	0.21
No	1.00	reference		1.00	reference	

Abbreviations: *NPC-RT* nasopharyngeal cancer patients with radiotherapy, *Non-NPC–RT* other head and neck cancer patients with radiotherapy, *Non-NPC-NRT* other head and neck cancer patients without radiotherapy, *HTN* hypertension, *DM* diabetes mellitus, *COPD* chronic obstructive pulmonary disease, *CKD* chronic kidney disease, *CAD* coronary artery disease, *HR* hazard ratio, *CI* confidence interval

The mortality proportion was 32.9, 55.8, and 21.7% in the NPC-RT, non-NPC-RT, and non-NPC-NRT groups, respectively (Table 1). Because of these high mortality proportion, an association between RT and intracranial aneurysms development could be inferred. We performed a competing risk analysis and this adjustment yielded the same results. The NPC-RT group having the highest risk (HR = 2.57, 95% CI: 1.55–4.24, *P* <  0.001) and the non-NPC-NRT group having the lowest risk (HR = 0.70, 95% CI: 0.32–1.56, *P* = 0.38) of intracranial aneurysm development. Meanwhile, hypertension was still associated with intracranial aneurysms formation (HR = 2.14, 95% CI: 1.34–3.43, *P* <  0.01).

## Discussion

Since intracranial aneurysms after RT was first reported in 1963, many studies have described this association. However, the majority of these were case studies without a comparison group, as the incidence of intracranial aneurysms after RT is low. Additionally, the diagnosis of radiation-induced intracranial aneurysms remains controversial due to a limited number of cases, the need for long-term follow-up after RT, a lack of statistical analysis, and the lack of established diagnostic criteria. NHIRD was established since 1996 and includes the medical data from 26 million population. In the present study, NHIRD was used to obtain a large sample size and the long-term follow-up to explore the potential relationship between RT and the incidence of intracranial aneurysms formation.

The incidence of radiation-induced intracranial aneurysms is affected by many factors and is thus difficult to determine [40]. In a study by Vernooij et al., the incidence was 1.8% [41]. Meanwhile, Omura et al. reported that 19% of patients studied had steno-occlusive changes of their cerebral arteries after external radiation [42]. Cappelli et al. also reported that 17% of patients had cerebrovascular complications after RT for optic tumors [43]. In this study, the incidence rate of intracranial aneurysms was 47.8 per 100,000 person-years in patients with NPC treated with RT. The incidence rate reported in the present study was much lower than that reported in previous studies.

This low incidence might be related to the strict criteria used in this study. First, the ICD-9 codes 4373 was used for intracranial aneurysm formation. Besides, there was no ICD-9 code for ruptured aneurysms. We used the ICD-9 procedure code for aneurysm clipping (3951) to enroll the ruptured or nonruptured aneurysm patients. Second, some patients with intracranial spontaneous subarachnoid hemorrhage (SAH) and were in a deep coma (Glasgow Coma Scale less than six) may not receive further imaging studies such as angiography or computed tomography (CT) angiography due to unstable vital signs. Therefore, these patients were not diagnosed with intracranial aneurysms. Third, a patient diagnosed with an aneurysm may be treated with endovascular coiling instead of surgical clipping in recent years. Unlike surgical clipping, no appropriate ICD-9 codes exist for coiling. Therefore, we were unable to include patients treated with coiling or other endovascular techniques in this study. Finally, patients who developed intracranial aneurysms with no symptoms did not undergo imaging studies, and were thus not identified. Together, these circumstances may have contributed to the under-estimation and low reported incidence rate of intracranial aneurysms formation in the present study. Despite the low incidence rate presented in this study, the risk of developing an intracranial aneurysm was higher in patients with NPC than in patients with non-NPC.

**Fig. 3:**
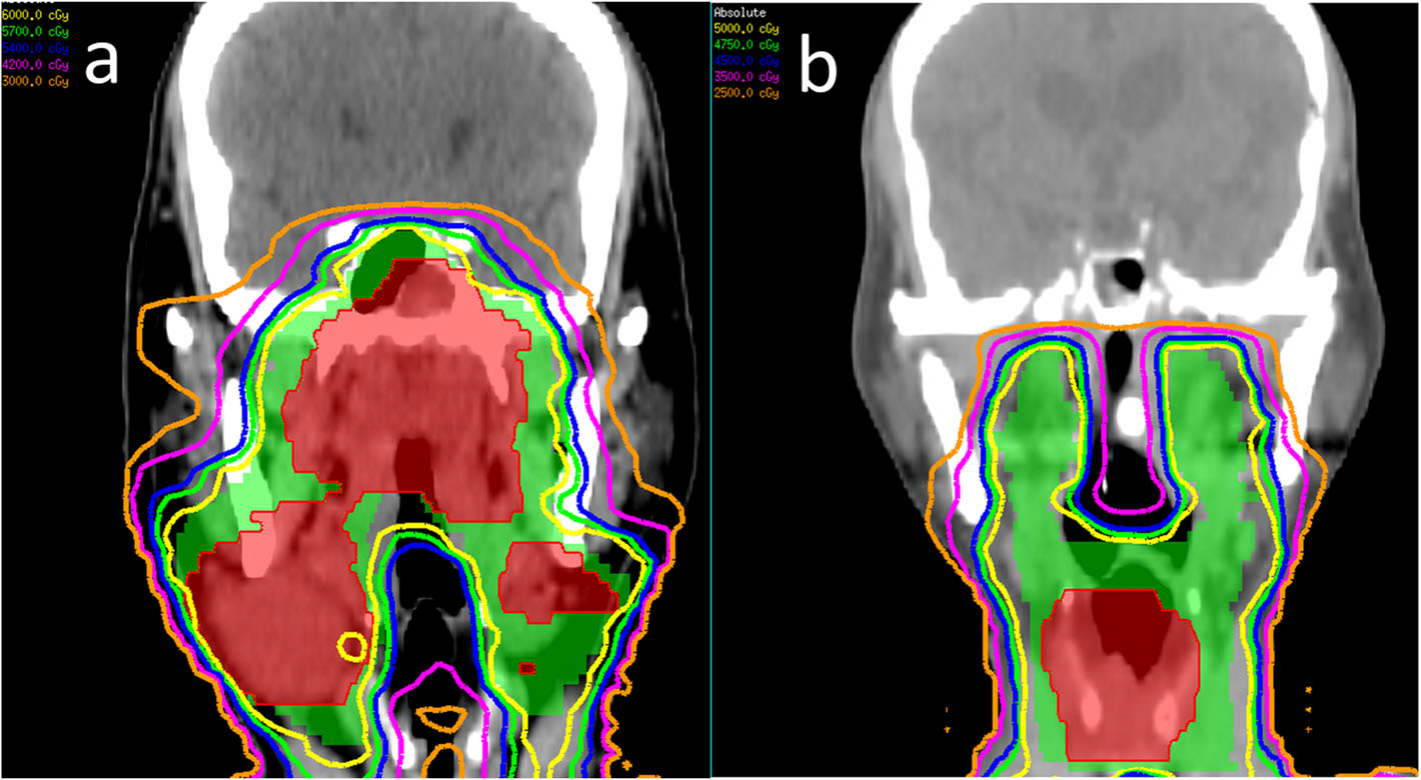
**a** Nasopharyngeal cancer, cT3N2M0, stage III. A 60-Gy dose was prescribed to the entire nasopharynx, posterior third of the nasal cavity and maxillary sinus, pterygoid fossae, parapharyngeal space, retropharyngeal lymph nodes, clivus, skull base, sphenoid sinus, and bilateral upper neck lymph nodes. A 70-Gy dose was prescribed to the gross tumor and lymphadenopathies areas (red area). **b** Supraglottic cancer, cT3N0M0, stage III. A 50-Gy dose was prescribed to the retropharyngeal lymph nodes and bilateral neck lymph nodes. A 70-Gy dose was prescribed to the gross tumor area (red area)

The underlying reason for this significantly higher risk of intracranial aneurysm formation in NPC patients receiving RT may be related to the higher radiation dose to intracranial vessels during RT (Fig. 3). The clinical target volume (CTV) in NPC patients includes the entire nasopharynx, posterior third of the nasal cavity, the maxillary sinus, pterygoid fossae, parapharyngeal space, retropharyngeal lymph nodes, clivus, skull base, sphenoid sinus, and bilateral neck lymph nodes [44, 45]. For other head and neck cancers, the CTV rarely included the clivus, skull base, and sphenoid sinus unless a tumor is involved. Therefore, the intracranial vessels of the majority of NPC patients received a high radiation dose, especially the vessels in the cavernous sinus. In contrast, few non-NPC patients received high radiation doses to the intracranial vessels.

The risk of intracranial aneurysm formation was slightly higher in the non-NPC-RT group compared with the non-NPC-NRT group, but this difference was not statistically significant. As mentioned above, the intracranial vessels of non-NPC-RT group patients usually received little or no radiation dose. However, some non-NPC-RT group patients may receive high radiation dose to intracranial vessels if they had advanced disease with tumor invasion to parapharyngeal space, infratemporal fossa or skull base. In addition, the radiation effect could potentially cause vascular abnormality for non-NPC-RT group patients receiving little radiation dose to intracranial vessels. This systematic effect of radiation has been described in previous studies [46–48]. Maduro et al. reported a risk of myocardial infarction in patients with cervical cancer after RT, and Nilsson et al. reported an increased risk of stroke in patients with breast cancer after RT.

The time interval needed to induce intracranial aneurysms formation after RT is another important issue for clinical physician. In previous studies, the time interval ranged from 6 months to 8 years [43, 49, 50]. In a literature review by Nanney et al., the average interval ranged from 5.71 years in a stereotactic radiosurgery (SRS) group to 11.24 years in a focused radiation group. They found that elderly individuals had a shorter interval than did younger patients. Thus, this time interval may be related to a higher level of atherosclerosis, a higher dose in a limited field, or a larger exposure field. The interval between RT and aneurysm formation in the present study was 4.3 ± 3.1 years; this was similar to intervals reported in other studies. However, in the non-NPC-RT group, the time interval was only 1.9 ± 2.2 years. This short interval may be related to the high mortality proportion (55.8%) in the non-NPC-RT group.

Malignancy-related death may lower the reported incidence of radiation-induced intracranial aneurysms. Therefore, competing risk analysis was used to more precisely explore the potential risk factors involved. After adjusting for risk of cancer-related death, patients in the NPC-RT group still had the highest risk of developing radiation-induced intracranial aneurysms (HR = 2.57, 95% CI: 1.55–4.24, *P* <  0.001). Patients with non-NPC who were treated with RT had a higher risk of developing intracranial aneurysms than did those who did not receive RT, but this difference was not statistically significant (HR = 0.70, 95% CI: 0.32–1.56, *P* = 0.38). Moreover, patients with hypertension had a higher risk of developing newly diagnosed intracranial aneurysms (HR = 2.14, 95% CI: 1.34–3.43, *P* <  0.01) and the association between hypertension and intracranial aneurysms formation has been well documented in other study [51]. Another interesting finding of this study was related to gender. Traditionally, females have been considered to have a higher risk of intracranial aneurysm formation [52]; however, males may have a higher risk of developing radiation-induced intracranial aneurysms [24]. We obtained similar results in our study, as the majority of patients who developed aneurysms was male. However, based on multiple regression analysis, male gender was not significantly associated with intracranial aneurysms formation (HR = 0.71, 95% CI: 0.39–1.28, *P* = 0.26).

There are several limitations to our study. First, RT techniques including two-dimensional RT, three-dimensional conformal RT or intensity modulated radiation therapy (IMRT) cannot be differentiated by using the NHIRD. Moreover, each patient may receive different radiation dose. However, total dose of 66–74 Gy in 1.8–2.12 Gy per fraction were usually prescribed for head and neck patients with gross tumor and total dose of 60–66 Gy in 1.8–2 Gy per fraction were usually prescribed for head and neck patients in adjuvant treatments. Therefore, the total radiation dose differences between NPC-RT and non-NPC-RT groups were limited. Consequently, we were able to compare the effect of radiation based only on the different radiation fields between these two groups. Next, we were unable to retrieve imaging data. Unlike general aneurysms, radiation-induced intracranial aneurysms can originate from the arterial wall rather than a branching site [13]. This uncommon location may be related to vessel wall degradation caused by radiation [24]. In addition to aneurysm location, the shape of an aneurysm may also be affected by RT. Radiation-induced intracranial aneurysms are more likely to be fusiform or pseudoaneurysms [40]. Because no detailed imaging data are included in the NHIRD, it was unable to confirm the locations and shapes of aneurysms in this study. Cahan et al. [53] mentioned that radiation-induced tumors should be located within the radiation field and that they should not present before radiation. While the systemic effects of radiation should be considered, detailed imaging reports are required to improve diagnostic accuracy. Finally, other risk factors of developing aneurysms formation including smoking, alcohol abuse and family history of intracranial aneurysms were not recorded in NHIRD [51, 54, 55]. It was unable to analyze the confounding effect of these well-known risk factors. Because of these limitations, more studies were needed to determine the diagnostic criteria of radiation-induced intracranial aneurysms.

## Conclusions

In the present study, NPC patients treated with RT were at higher risk of developing intracranial aneurysms than were patients with non-NPC, when treated with or without RT. Moreover, the low incidence of RT-induced intracranial aneurysms in this study was under estimation and the real number would be higher. While considering the catastrophic outcome after intracranial aneurysm rupture, the long-term follow-up is warranted if patients have a history of high radiation dose exposure to intracranial vessels.

## Data Availability

The datasets generated during and analysed during the current study are available from the corresponding author on reasonable request.

## Abbreviations

CAD: Coronary artery disease
CI: Confidence interval
CKD: Chronic kidney disease
CT: Computed tomography
CTV: Clinical target volume
HR: Hazard ratio
IMRT: Intensity modulated radiation therapy
NHIRD: The National Health Insurance Research Database
NPC: Nasopharyngeal carcinoma
RT: Radiotherapy
SAH: Subarachnoid hemorrhage
SRS: Stereotactic radiosurgery
